# Ewing's Sarcoma of the Kidney Complicated by a Wunderlich Syndrome

**DOI:** 10.1155/2015/601038

**Published:** 2015-04-01

**Authors:** Mihai Razvan Manescu, Achraf Sahyoun, Nicolas Froment, Nicolae Crisan, Vincent Girot

**Affiliations:** ^1^Department of Urology, Mercy Hospital Metz-Thionville, allée du Château, 57530 Ars-Laquenexy, France; ^2^Department of Anatomical Pathology, Mercy Hospital Metz-Thionville, allée du Château, 57530 Ars-Laquenexy, France; ^3^Department of Urology, Municipal Clinical Hospital Cluj-Napoca, Tabacarilor 11, 400139 Cluj-Napoca, Romania

## Abstract

The Wunderlich syndrome found after the rupture of primitive renal Ewing's sarcoma is not a situation that we find often in everyday practice. The clinical findings are not specific, which is why the differential diagnosis must be made with a multitude of benign and malignant renal masses until the correct diagnosis can be made by the pathologist. The CT and MRI images are not characteristic. One treatment option is the multidisciplinary approach; however, the prognosis remains poor for patients with metastatic disease.

## 1. Introduction

A Wunderlich syndrome caused by the rupture of Ewing's sarcoma of the kidney is a rare entity and represents a life-threatening emergency. In the beginning, PNET (primitive peripheral neuroectodermal tumours) and Ewing's sarcoma were considered different but today, because of their similar histologic and cytogenetic characteristics, they are included in the same family with Ewing's sarcoma.

## 2. Case Report

**Figure 1:**
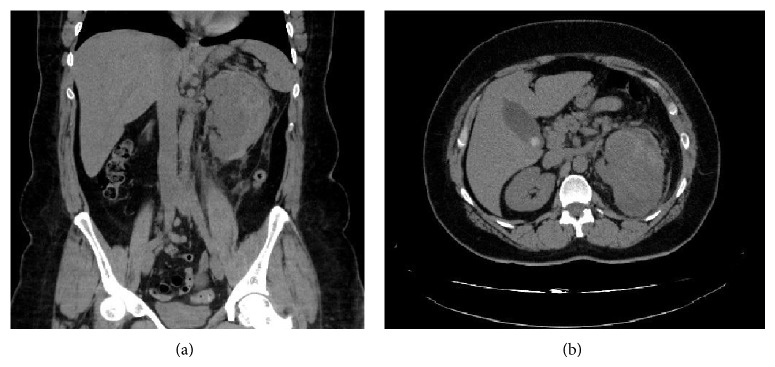
(a) The coronal reconstructed CT image shows a large solid heterogeneous mass without calcifications in the left kidney and also a perirenal hematoma. (b) The axial CT image of the upper abdomen reveals a large solid heterogeneous mass measuring 7 × 6.7 in the left kidney and also a perirenal hematoma.

**Figure 2:**
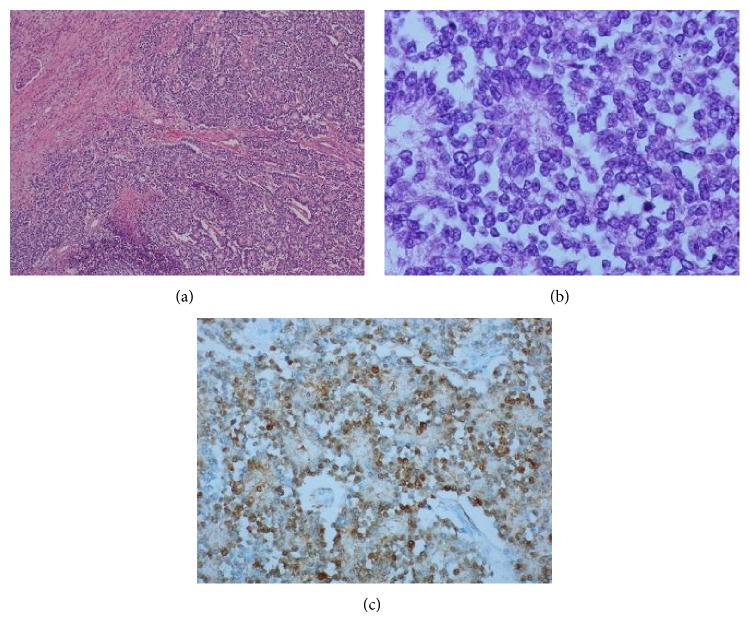
(a) Small blue cells arranged in Homer-Wright rosettes; (b) tumor cells and rosettes, numerous atypical mitoses; (c) tumor cells labeled with anti-S100 protein antibody.

A 40-year-old woman presented to the emergency room for left flank pain lasting for 2 hours and irradiating to the groin. Her medical and surgical history included aortic regurgitation, left kidney stones, stress urinary incontinence surgically treated, and obesity (BMI = 40.8). On physical examination, the patient was found to be afebrile and in good clinical status except for a positive Giordano's sign. Her blood haemoglobin was 12.7 g/dL with a normal coagulation profile and normal renal function. She was first treated with anti-inflammatory agents, which lead to a decrease of the clinical symptoms. A computed tomography scan showed a large solid heterogeneous mass measuring 7 × 6.7 cm, without calcifications, located in the left kidney, as well as a perinephric hematoma (Figure 1). No other secondary lesions were found at this time. The patient underwent left nephrectomy and the renal mass was later diagnosed as Ewing's sarcoma. On the histological sections, we found small round blue cells arranged in a nodular pattern and also in Homer-Wright rosettes (Figure 2). Vascular invasion was also observed. The immunohistochemistry tests revealed that the tumoral tissue was positive for CD99, P100, and C-Kit. The tumor cell proliferation rate assessed by the Ki-67 antigen-labeling index was above 50%. A PET-scan performed after the surgical intervention found a metastatic lesion in the left femoral head, which required surgical resection. Reconstruction was achieved by an endoprosthesis. The patient also underwent six cycles of VIDE: Oncovin, Holoxan, Mesna, Adriamycin, and Vepesid. Follow-up computed tomography scans showed no evidence of disease after 27 months.

## 3. Discussions

Ewing's sarcoma is a rare primary malignant renal tumor and it is characterized by an aggressive biological behaviour. The first Ewing's sarcoma was reported by Mor and colleagues. Later, John Ewing presented this type of tumor in the diaphysis of long bones [1]. The clinical findings are nonspecific and may include flank or abdominal pain, palpable mass, and hematuria, in decreasing order [2]. The tumor is more frequent in the male population (1.5 : 1) [3] and most of the patients are young adults, with median age 28 years (range 4–69 years) [2]. Usually the patients remain asymptomatic until the tumor is big enough to produce symptoms. The size of the tumor at the moment of diagnosis varies in the literature from 5.5 to 23 cm. The CT and MRI characteristics can also be found in the case of other types of renal tumor [4]. Most of the renal masses incidentally discovered are benign renal cysts but the differential diagnosis should also be made with abscesses, lymphomas, metastases from a distant primary lesion, oncocytomas, renal adenomas, sarcomas, Wilms tumors, and renal cell carcinomas. The final diagnosis is made by the pathologist. Macroscopically the tumor is tan-white with areas of hemorrhage and necrosis. The microscopic examination shows a monomorphic population of small blue cells that can be arranged in Homer-Wright rosettes. Molecular and immunohistochemistry studies are needed for the histological differential diagnosis with a Wilms tumor and a lymphoma, metastatic neuroblastoma, rhabdomyosarcoma, synovial sarcoma, or small cell carcinoma [2]. Usually Ewing's sarcoma associates an overexpression of the CD99 membrane protein and expression of the friend leukemia virus integration (FLI-1) [2]. Approximately 85–90% of Ewing's sarcomas associate a functional oncogene resulting from the DNA translocation t(11;22)(q24;q12) [5]. Because the treatment is complex and includes surgery, chemotherapy, and radiotherapy, a multidisciplinary approach is recommended [6].

The Wunderlich syndrome was described for the first time in 1856 as a spontaneous renal bleeding confined to the perinephric or subcapsular space [7]. It is a rare entity and represents a life-threatening emergency. Clinically the patient could present lumbar pain, general status deterioration, and also a palpable mass. The most frequent causes include benign and malignant renal neoplasms: oncocytoma, renal cell carcinoma, and angiomyolipoma. To the best of our knowledge, there is so far no data in the published literature referring to the Wunderlich syndrome as a form of clinical presentation for Ewing's renal tumour. The method of choice to identify a Wunderlich syndrome, with a sensitivity of 100%, is the CT scan. The treatment should be chosen depending on the cause either surgery or a more minimally invasive technique: selective renal arterial embolization [8]. In the discussed case, given the emergency, we preferred the surgical intervention with a therapeutic, as well as a diagnostic, role.

Ewing's sarcomas have obvious metastases at the time of diagnosis in 20–25% of the cases, being the main indicator of prognosis. Furthermore, after local therapy, 80–90% of the cases develop in time systemic relapse, suggesting that the metastases had been present subclinically at the moment of diagnosis [4]. The most common sites of secondary determinations of Ewing's sarcoma are the liver, lungs, and the skeletal system. The use of advanced imaging methods (PET-scan) allowed us to objectify and find a solution for the metastatic disease, therefore improving the prognosis: the patient was relapse-free after 27 months.

## 4. Conclusions

Wunderlich syndrome after the rupture of a renal Ewing's sarcoma is a rare entity, which represents a diagnostic challenge and already aggressive tumor. The CT and MRI images are not specific that is why the true diagnosis will be made by the anatomopathologist after molecular, immunohistochemistry, and histologic tests. It is important to have a definite diagnosis in order to choose a good multimodal treatment, due to the tumor's poor prognosis and to the young age of most of the patients.
